# Reproduction of an azooxanthellate coral is unaffected by ocean acidification

**DOI:** 10.1038/s41598-017-13393-1

**Published:** 2017-10-12

**Authors:** Francesca Gizzi, Ludovica de Mas, Valentina Airi, Erik Caroselli, Fiorella Prada, Giuseppe Falini, Zvy Dubinsky, Stefano Goffredo

**Affiliations:** 1 Marine Science Group, Department of Biological, Geological and Environmental Sciences, University of Bologna European Union, Via F. Selmi 3, I-40126 Bologna, Italy; 2 Department of Chemistry “Giacomo Ciamician”, University of Bologna European Union, Via F. Selmi 2, I-40126 Bologna, Italy; 3 The Mina and Everard Goodman Faculty of Life Sciences, Bar-Ilan University, Ramat-Gan, Israel

## Abstract

Anthropogenic carbon dioxide (CO_2_) emissions and consequent ocean acidification (OA) are projected to have extensive consequences on marine calcifying organisms, including corals. While the effects of OA on coral calcification are well documented, the response of reproduction is still poorly understood since no information are reported for temperate corals. Here we investigate for the first time the influence of OA on sexual reproduction of the temperate azooxanthellate solitary scleractinian *Leptopsammia pruvoti* transplanted along a natural *p*CO_2_ gradient at a Mediterranean CO_2_ vent. After 3 months, future projection of pH levels did not influence the germ cell production, gametogenesis and embryogenesis in this azooxanthellate coral. These findings suggest that reproductive potential may be quite tolerant to decreasing pH, with implications for ecosystem function and services in a changing ocean.

## Introduction

Anthropogenic CO_2_ absorbed by the ocean is decreasing seawater pH and changing ocean chemistry, reducing the availability of carbonate ions (CO_3_^2−^)^1^, the building blocks used by calcifying marine organisms, such as corals^2^. Seawater pH has already decreased by 0.1 pH units since the industrial revolution^1,3^ and if CO_2_ emissions continue under the “business-as-usual” scenario, oceanic pH levels will drop to 7.8 by the end of the century and may reach 7.4 units by 2500^1,3^.

Several studies reveal how calcifying algae^4,5^, corals^4,6,7^ and coral reef communities, including fish^8^, show reduced calcification rates under low seawater pH, due to depleted carbonate saturation. Moreover, many biological processes and physiological functions independent of calcification may be impacted by decreasing pH^9–11^. Ocean acidification negatively influences the metabolism of marine invertebrates that have a low ability to compensate for disturbances to the extracellular ion and acid-base status, leading to negative effects on reproduction and behaviour^12^. Sexual reproduction represents a crucial process in the development and persistence of populations and its reduction threatens the resilience of species, leading to shifts in size and abundance of populations^13^. Early life history stages of echinoderms, mollusks and crustaceans including larval availability^9–11,14–19^ (gamete production, fertilization etc.), larval development^9–11,14–16,20^ and growth^14,20^, larval settlement^9,20^, post-settlement growth^11^ and survival^11^ seem to be very sensitive to increasing *p*CO_2_. The few available studies on the effects of ocean acidification on coral sexual reproduction were conducted in tropical species and only under laboratory conditions^19,21–24^, thus lacking information on the complex natural environment^25^.

Naturally acidified areas (i.e. CO_2_ vents) mimic future ocean conditions and can be used as natural laboratories to investigate the effects of increasing *p*CO_2_ on marine ecosystems *in situ* ^6,26–29^. The Panarea CO_2_ vent (Tyrrhenian Sea, Italy) is shallow, and characterized by almost pure CO_2_ emissions at ambient temperature, with no toxic compounds, making this an ideal site for ocean acidification studies in a natural setting^28,29^. The *p*CO_2_ gradient is characterized by extremely low pH values on the rim of the crater that exceed the most pessimistic scenarios for the coming century, representing a more distant scenario (2500^1,3^) and by progressively higher values that match near future scenarios (2100) all the way to the periphery where normal pH conditions are re-established.

This study investigated for the first time at the Panarea CO_2_ vents the effects of future pH scenarios on coral reproductive efficiency. The model species is a solitary, azooxanthellate, gonochoric and brooding^30^ Mediterranean scleractinian, *Leptopsammia pruvoti*, Lacaze-Duthiers^31^ (Supplementary Fig. S1). A previous study on reproductive cycle of this species showed that its spermatogenesis follows an annual cycle, while the oogenesis is characterized by a 2-year cycle. From November to January the size of reproductive elements increases, fertilization takes place from January to April, and planulation occurs in May and June^32^.

Previous studies on *L. pruvoti* reported that sea surface temperature and solar radiation do not affect its skeletal bulk density^33^, skeletal porosity^33^, population abundance^34^ and structure stability^35^, net calcification rate^36^ and reproduction^37^ along an 850-km latitudinal gradient on the west coast of Italy. Specimens of *L. pruvoti* transplanted along the same *p*CO_2_ gradient as in the present study show a decrease in net calcification rates with decreasing pH, and a significant increase in polyp mortality rate, but only when average temperatures is high^38^.

## Results

Gametogenetic polyps together with sexually inactive individuals (without germ cells), were found in both, the gonadal development and fertilization periods (Supplementary Table S1).

**Figure 1:**
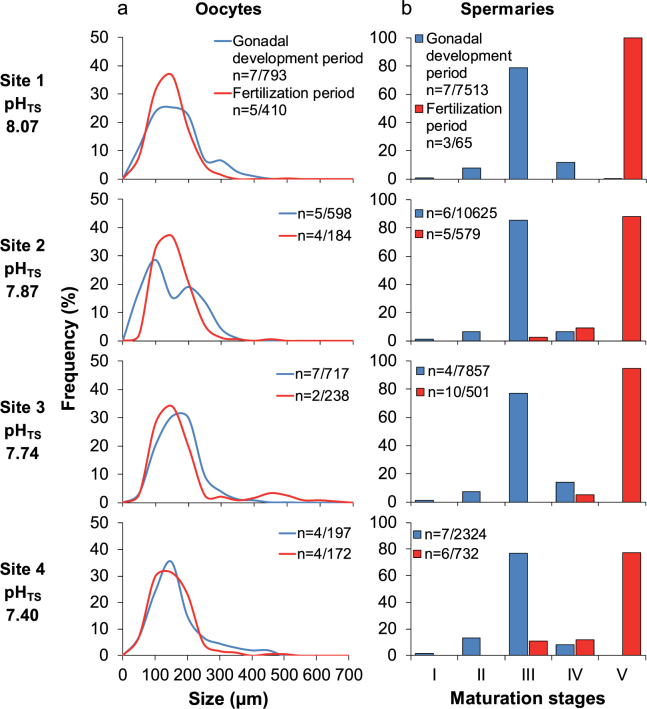
Oocyte size and spermary maturation stage distributions. (**a**) Distribution of oocyte size during gonadal development (blue line) and fertilization (red line) periods. n = number of polyps/oocytes. (**b**) Distribution of the five maturation stages of spermaries during gonadal development (blue histogram bars) and fertilization (red histogram bars) periods. n = number of polyps/spermaries.

Size/frequency distribution of oocytes and maturation stage/frequency distribution of spermaries were significantly different between gonadal development period and fertilization period, in all sites (Kolmogorov-Smirnov test, oocyte: p < 0.001 for site 1 *vs* 1 and for site 3 *vs* 3, p = 0.002 for site 2 *vs* 2, p = 0.009 for site 4 *vs* 4, spermary: p < 0.001 for all sites; Fig. 1a,b).

### Gonadal development period

**Figure 2:**
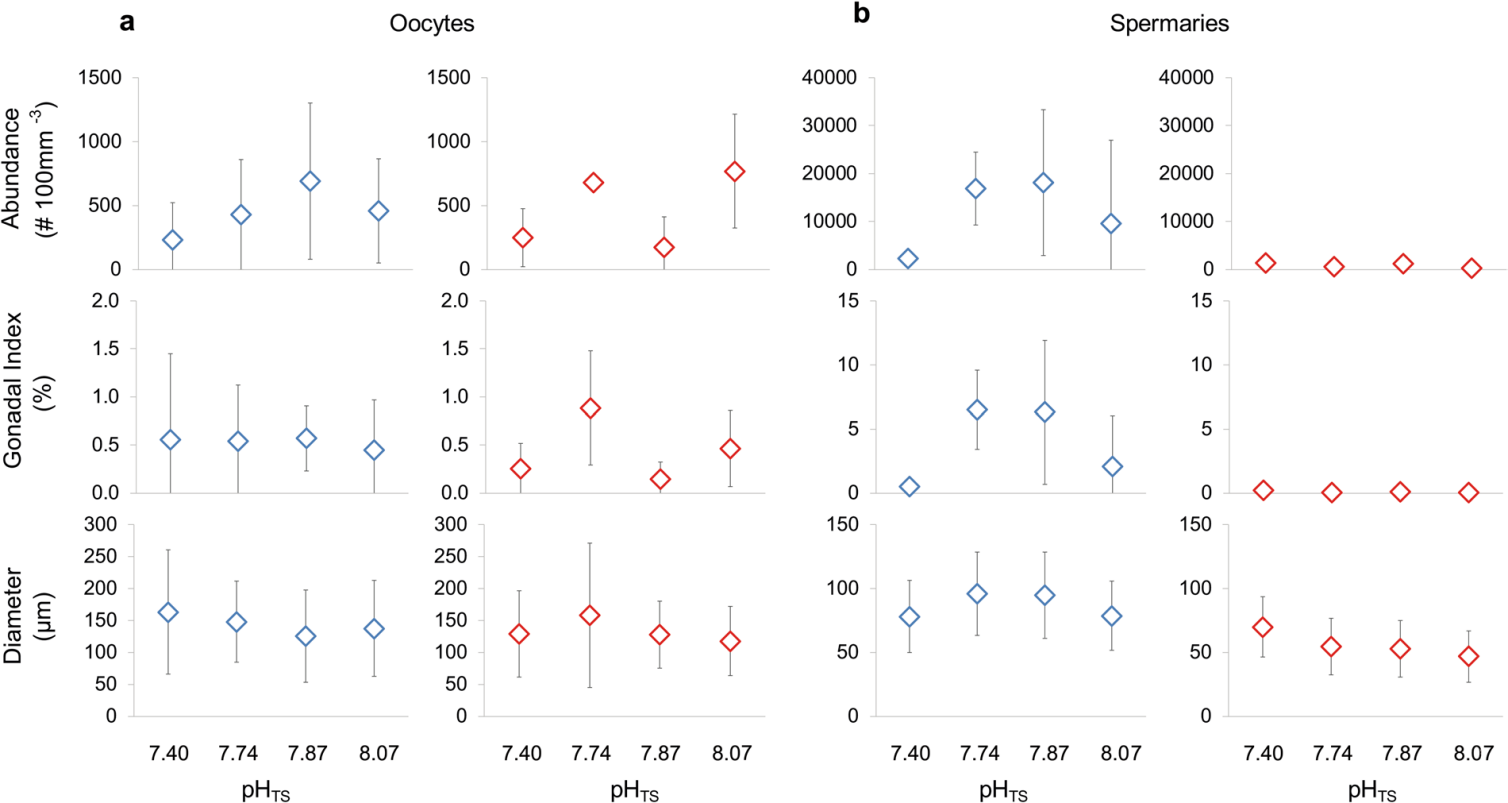
Oocyte and spermary reproductive parameters. Mean ± standard deviations of abundance, gonadal index and diameter of (**a**) oocytes and (**b**) spermaries in the gonadal development (blue dots) and fertilization (red dots) periods. Number of samples and mean values for each site and period are listed in Table 1 for oocytes and in Table 2 for spermaries.

Oogenesis was characterized by a cohort of small oocytes in all sites, without difference in size/frequency distribution among sites (PERMANOVA, p = 0.293; Fig. 1a). Spermatogenesis showed small spermaries with the mode in developmental stage III at all sites and homogeneous distributions among sites (PERMANOVA, p = 0.443; Fig. 1b). Abundance, gonadal index and diameter of both, oocyte and spermary, did not show differences among sites (Kruskal-Wallis test with Monte Carlo estimate, oocyte abundance p = 0.486 and gonadal index p = 0.795, spermary abundance p = 0.152 and gonadal index p = 0.061; PERMANOVA, oocyte diameter p = 0.892 and spermary diameter p = 0.051; Tables 1 and 2; Fig. 2a,b

**Table 1:** Oocyte reproductive parameters at the four sites in gonadal development and fertilization periods.

Site	pH_TS_	n_p_	Abundance (# 100 mm^−3^)	Gonadal Index (%)	n_o_	Diameter (µm)
**Gonadal development period**						
1	8.07	7	457 ± 302	0.45 ± 0.39	793	138 ± 5
2	7.87	5	690 ± 536	0.57 ± 0.30	598	126 ± 6
3	7.74	7	429 ± 319	0.54 ± 0.43	717	147 ± 5
4	7.4	4	233 ± 286	0.56 ± 0.88	197	163 ± 14
P			NS 0.486	NS 0.795		NS 0.892
**Fertilization period**						
1	8.07	5	768 ± 390	0.46 ± 0.35	410	118 ± 5
2	7.87	4	173 ± 234	0.14 ± 0.18	184	128 ± 8
3	7.74	2	681 ± 91	0.88 ± 0.82	238	158 ± 14
4	7.4	4	250 ± 222	0.25 ± 0.26	172	129 ± 10
P			NS 0.112	NS 0.267		NS 0.192

Abundance, gonadal index and diameter values are shown as mean ± CI 95%. n_p_ = polyps number; n_o_ = oocytes number. P = p value of Kruskal-Wallis equality-of-populations rank test with the Monte Carlo estimate for abundance and gonadal index and PERMANOVA for diameters. NS = not significant.

**Table 2:** Spermary reproductive parameters at the four sites in gonadal development and fertilization periods.

Site	pH_TS_	n_p_	Abundance (# 100 mm^−3^)	Gonadal Index (%)	n_s_	Diameter (µm)
**Gonadal development period**						
1	8.07	7	9646 ± 12790	2.06 ± 2.97	7513	79 ± 1
2	7.87	6	18139 ± 12181	6.31 ± 4.49	10625	95 ± 1
3	7.74	4	16941 ± 7464	6.52 ± 3.02	7857	96 ± 1
4	7.4	7	2232 ± 1664	0.50 ± 0.36	2324	78 ± 1
P			NS 0.152	NS 0.061		NS 0.051
**Fertilization period**						
1	8.07	3	293 ± 387	0.02 ± 0.02	65	47 ± 5
2	7.87	5	1167 ± 796	0.08 ± 0.07	579	53 ± 2
3	7.74	10	596 ± 373	0.05 ± 0.04	501	55 ± 2
4	7.4	6	1353 ± 326	0.20 ± 0.19	732	70 ± 2
P			NS 0.159	NS 0.176		NS 0.521

Abundance, gonadal index and diameter values are shown as mean ± CI 95%. n_p_ = polyps number; n_s_ = spermaries number. P = p value of Kruskal-Wallis equality-of-populations rank test with the Monte Carlo estimate for abundance and gonadal index and PERMANOVA for diameters. NS = not significant.

).

### Fertilization period

**Figure 3:**
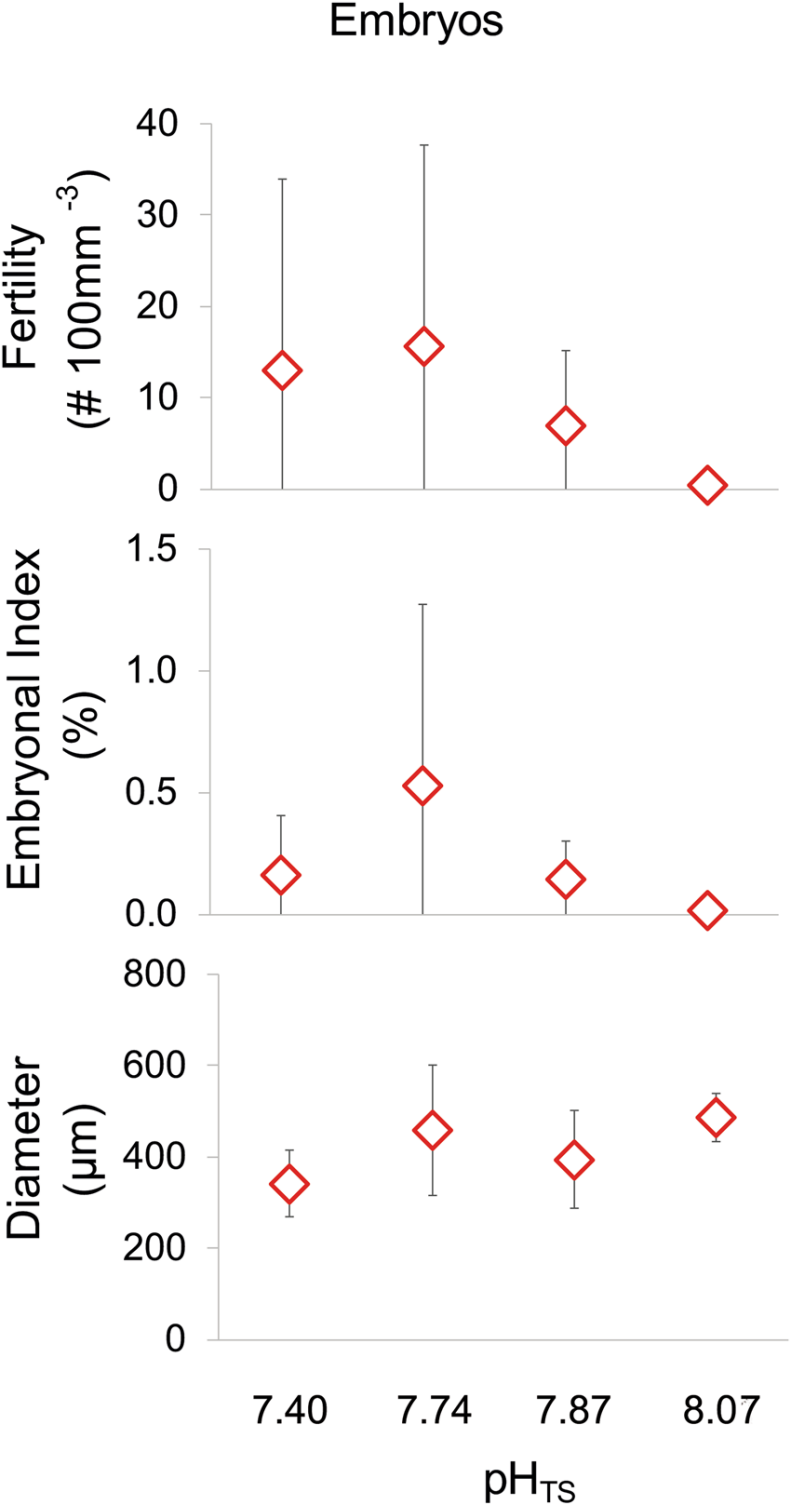
Embryo reproductive parameters. Mean ± standard deviations of fertility, embrional index and embryo diameter in the fertilization period. Samples number and mean values for each site are listed in Table 3.

Most of the oocytes were smaller than 300 µm at all sites without differences in size/frequency distribution among sites (PERMANOVA, p = 0.676; Fig. 1a). Spermatogenesis was characterized by advanced maturation stages in all sites and no differences were found among sites (PERMANOVA, p = 0.379; Fig. 1b). Oocyte and spermary abundance, gonadal index and diameter were homogeneous among sites (Kruskal-Wallis test with Monte Carlo estimate, oocyte abundance p = 0.112 and gonadal index p = 0.267, spermary abundance p = 0.159 and gonadal index p = 0.176; PERMANOVA, oocyte diameter p = 0.192 and spermary diameter p = 0.521; Tables 1 and 2; Fig. 2a,b). During this period, female polyps containing embryos in the coelenteric cavity were found at all sites, and fertility, embryonal index and embryo diameter did not differ along the gradient (Kruskal-Wallis test with Monte Carlo estimate, p = 0.819 for fertility and p = 0.796 for embryonal index; PERMANOVA, p = 0.083 for embryo diameter; Table 3; Fig. 3

**Table 3:** Embryo reproductive parameters at the four sites in the fertilization period.

Site	pH_TS_	n_p_	Fertility (# 100 mm^−3^)	Embrional Index (%)	n_e_	Diameter (µm)
**Fertilization period**						
1	8.07	5	0.4 ± 0.6	0.02 ± 0.03	6	486 ± 43
2	7.87	4	7.0 ± 8.0	0.14 ± 0.16	27	395 ± 40
3	7.74	2	15.6 ± 30.6	0.53 ± 1.03	17	459 ± 68
4	7.4	4	13.0 ± 20.6	0.16 ± 0.24	23	341 ± 30
P			NS 0.819	NS 0.796		NS 0.083

Fertility, embrional index and diameter values are shown as mean ± CI 95%. n_p_ = polyps number; n_e_ = embryos number. P = p value of Kruskal-Wallis equality-of-populations rank test with the Monte Carlo estimate for fertility and embrional index and PERMANOVA for diameters. NS = not significant.

).

## Discussion

This is the first detailed study concerning the effect of high *p*CO_2_ on the reproductive output of corals transplanted along a natural *p*CO_2_ gradient.

Increasing *p*CO_2_ showed no effects on the production of male and female germ cells, as suggested by the oocyte and spermary abundances, which were homogenous along the gradient in both periods analyzed, and by the presence of embryos in female corals during the fertilization period at all sites.

During both, gonadal development and fertilization period, the development of male and female germ cells did not follow trends associated with increasing *p*CO_2_ (Fig. 1a,b), and all reproductive parameters of oocytes and spermaries seemed unrelated to decreasing pH, suggesting that this species could be quite tolerant to acidified conditions (Fig. 2a,b). These results are in agreement with previous studies performed in aquaria. After 6 months under pH levels projected for the end of this century, the tropical zooxanthellate coral *Montipora capitata* showed no effect on gametogenesis and gamete production^22^. The same result was found in *Oculina patagonic*a after 12 months under similar pH conditions^21^. A negative effect was found in the sea urchin *Strongylocentrotus droebachiensis*, which after 60 days under high *p*CO_2_ conditions (average pH 6.98), shows a significant decrease in gonadal development^17^.

During the fertilization period, the detection of embryos at all sites suggested that the fertilization process had started at all sites. Moreover, no differences were found in the embryo production and embryo size, indicating that after three months under experimental conditions, also the fertilization process and embryogenesis of *L. pruvoti* were unaffected by future *p*CO_2_ levels.

Influence of decreasing pH on fertilization has been well studied through aquarium experiments in marine invertebrates, such as echinoderms, mollusks, crustaceans and tropical corals. Many experiments show negative responses, especially if sperm concentration is low and limited^11,19,20,39^. Fertilization success of the tropical coral *Acropora palmata* decreases with increasing *p*CO_2_, with a reduction (averaged across all sperm concentrations) of 13% at the CO_2_ level expected for the end of this century^23^. At the contrary, the fertilization process of *A. tenuis* and *A. millepora* is unaffected by elevated *p*CO_2_ projected for the end of this century, alone or in combination with temperature^40^, but only if sperm concentration is high^41^.

Moreover, experimental laboratory studies show that increasing *p*CO_2_ negatively affects embryo growth and development in mollusks^9,14^, echinoderms^15,16,39,42^ and crustaceans^43^, but not significant effect was detected on the timing of embryonic development in the tropical corals *A. palmata* under seawater pH levels expected for the end of this century (pH 7.7^44^).

The solitary non-zooxanthellate *L. pruvoti* seemed quite tolerant to future pH levels, showing normal reproduction along the gradient. The lack of effects on gametogenesis and embryogenesis in *L. pruvoti* under decreasing pH may be explained in different ways.

The gametogenesis of *L. pruvoti* extends over the year, in fact, male germ cells takes approximately 12 months to mature while female germ cells need ~24 months^32^. Considering the short-term exposure to experimental conditions in this study (3 months), spermatogenesis was exposed to high *p*CO_2_ levels for a quarter of its time, while oogenesis was exposed for one eighth of its length, possibly explaining the low sensitivity observed with future pH levels.

Another explanation concern the channeling of available energy into reproduction at the expense of other metabolic processes. The high investment of energy that *L. pruvoti* places to keep the gametogenesis constant along the pH gradient may leave little energy for the coral to sustain net calcification in the face of ocean acidification^45^. Confirming this hypothesis, a parallel study on *L. pruvoti* transplanted along the same natural *p*CO_2_ gradient shows decreased net calcification rate with decreasing pH^38^, even under pH values projected for the end of this century. Thus, under future pH conditions, *L. pruvoti* may allocate more energy to maintain constant the reproduction process, at the expense of net calcification, which significantly decreases under acidified conditions.

In conclusion, the reproductive process of *L. pruvoti* seems that will be fine in coming decades, showing no effects on gametogenesis, spermatogenesis and embryogenesis along the pH gradient. However, this study did not consider the post-fertilization process, including settlement success, larval survival and development and juvenile growth, even if other studies have reported significant impact of ocean acidification on tropical corals.

This study considered the short-term effect of pH (~3 months), but further investigations are needed to understand if *L. pruvoti* is capable of maintaining a constant reproductive output, fertilization success and embryo growth under a long-term exposure to future pH levels.

## Materials and Methods

### Study site

The experimental site, which has been previously described in detail by Goffredo *et al*.^28^, is located near Panarea Island (Mediterranean Sea, Aeolian Archipelago, Italy, 38°38′16″N 15°06′37″E), where an underwater crater (20 × 14 m) at 10 m depth, generates a stable and continuous column of bubbles (98–99% CO_2_^28,29,46^) at ambient temperature, creating a natural pH gradient. Along this gradient, four sampling sites were selected: the control site (site 1: mean Total Scale (TS) pH_TS_ 8.07), located about 34 m away from the center of the crater, two intermediate pH sites (site 2 and 3: mean pH_TS_ respectively 7.87 and 7.74), representing the intermediate and the most pessimistic IPCC scenario for the end of this century and the extreme pH site (site 4: mean pH_TS_ 7.40), situated in proximity of the vents, representing the projection for 2500. The experimental site has stable hydrothermal–chemical properties and only *p*CO_2_ concentration differed significantly among the four sites (see Supplementary information of ^38^ for detailed seawater carbonate chemistry in each transplantation site).

### Ethics statement

This study was carried out following the fundamental ethical principles. According to the European normative, there is no active conservation measure for the Mediterranean scleractinian species under study (*L. pruvoti*). The species is not protected in Italy, nor is it subject to any regulations. Thus, no permit was needed to sample specimens. For this study, sampling was limited strictly to the number necessary for the described analyses and performed where the species has high population density to minimize the impact of removing individuals and preserve both the demographic and genetic structure of the natural populations.

### Sampling and field transplantation

Sexually mature *L. pruvoti* specimens (length > 3 mm, the size at sexual maturity of this species^32^) of similar size (average length 7 mm) were sampled at ~6 m depth by SCUBA diving in an area ~2 km away from the vent area and transplanted to the four sites in six experimental periods.

The same number of corals was randomly assigned to each of the four sites and each polyp was considered as a replicate (n = 4–6 polyps per site, per experimental period; Supplementary Table S1). Polyps were glued with a bicomponent epoxy coral glue (Milliput, Wales, UK) onto ceramic tiles and placed upside-down under plastic cages to mimic their natural orientation in overhangs and caves. Corals were exposed to experimental conditions during six different transplantation periods (September 2010–November 2010; November 2010–March 2011, March–June 2011, June–July 2011, July–December 2011 and April–June 2012) to identify the key moments and the seasonality of reproductive processes^32^. At the end of each transplantation period, samples were fixed in a formaldehyde fixative solution (10% formaldehyde and 90% seawater, saturated with calcium carbonate) to preserve the sample tissue for further histological analysis.

### Biometric and cyto-histometric analyses

Polyp length (L, maximum axis of the oral disk), width (w, minimum axis of the oral disk), and height (h, oral-aboral diameter) were measured for each specimen with a pair of calipers (±0.05 mm). The body volume (V) was estimated using the equation:

1 $${\rm{V}}=(L/2)\ast (w/2)\ast {\rm{h}}\ast {{\rm{\pi }}}^{32}$$

Biometric measurements were used to calculate the reproductive parameters (see paragraph below).

Polyps were post-fixed in Bouin solution. After decalcification in EDTA and dehydration in a graded alcohol series from 80 to 100%, polyps were embedded in paraffin, and serial transverse sections were cut at 7 µm intervals along the oral-aboral axis, from the oral to the aboral poles. Tissues were stained with Mayer’s hematoxylin and eosin^32^.

Cytometric analyses were performed with a light microscope NIKON Eclipse 80i using an image analysis software: NIKON NIS-Elements D 3.1. The maximum and minimum diameters of oocytes in nucleated sections, and spermaries, classified in five maturation stages in accordance with previous studies on gametogenesis of this species^30,32,37^, were measured. The maximum and minimum diameters of embryos found in the gastrovascular cavity were measured.

### Reproductive parameters

Reproductive output was defined by six parameters: 1) *oocyte* and *spermary abundance*, both defined as the number of reproductive elements per body volume unit (100 mm^3^); 2) *gonadal index*, defined as the percentage of body volume occupied by oocytes and spermaries; 3) *reproductive element size*, defined as the average of the maximum and minimum diameter of spermaries and oocytes in nucleated section; 4) *fertility*, defined as the number of embryos per body volume unit (100 mm^3^); 5) *embryonal index*, defined as the percentage of body volume occupied by embryos; 6) *embryo size*, defined as the average of the maximum and minimum diameter of embryos.

Based on the developing stages of oocytes and spermaries found in the analyzed samples and on the presence/absence of embryos in the coelenteric cavity of female polyps, samples from the six transplantation periods were grouped in two gonadal activity periods: 1) *gonadal development period*, characterized by the beginning of spermary and oocytes development, and 2) *fertilization period*, characterized by a little stock of big mature oocytes not yet fertilized, a bigger stock of oocytes that will be fertilized in the following reproductive year, advanced maturation stages of spermaries and presences of embryos in the coelenteric cavity of females.

### Statistical analyses

Levene’s test was used to test homogeneity of variance and one-sample Kolmogorov-Smirnov’s test was used to test normality of data distribution. When the sample size was lower than 2000, the Shapiro-Wilk test was used. The two-sample Kolmogorov-Smirnov’s test was used to do a pairwise comparison of the distribution of oocyte size and spermary maturation stages between the two periods. The Monte Carlo method^47^ solves problems in the non-parametric test for small samples, estimating the P-value by taking a random sample from the reference set and studies its permutations^48^ (10,000 random permutations in this study). This method was used to estimate the significance of the Kruskal-Wallis test when comparing the reproductive parameters (oocyte and spermary abundance and gonadal index; fertility and embryonal index) among sites.

The analyses were computed using PASW Statistics 22.0.

A one-way permutation multivariate analysis of variance (PERMANOVA)^49^ based on Euclidean distances was performed with 999 permutation, to test differences in oocyte size distribution, spermary maturation stage distribution and in oocyte, spermary and embryo diameter among sites, using the software Primer 6 (Quest Research Limited).

### Data availability

The datasets analysed during the current study are available from the corresponding author on reasonable request.

## Electronic supplementary material

Supplementary information
