# Supplementary material for: Reproduction of an azooxanthellate coral is unaffected by ocean acidification
Source: Sci Rep. 2017 Oct 12;7:13049. doi: 10.1038/s41598-017-13393-1 (PMC5638904; doi:10.1038/s41598-017-13393-1)
Supplement: Supplementary file 1 — Supplementary information [file 41598_2017_13393_MOESM1_ESM.pdf]

Supplementary information

Reproduction of an azooxanthellate coral is unaffected by ocean acidification

Francesca Gizzi[1], Ludovica de Mas[1], Valentina Airi[1], Erik Caroselli[1], Fiorella Prada[1], Giuseppe Falini[2], Zvy Dubinsky[3], Stefano Goffredo[1*]

[1]Marine Science Group, Department of Biological, Geological and Environmental Sciences, University of Bologna, Via F. Selmi 3, I-40126 Bologna, Italy, European Union.

[2]Department of Chemistry "Giacomo Ciamician", University of Bologna, Via F. Selmi 2, I-40126 Bologna, Italy, European Union.

[3]The Mina and Everard Goodman Faculty of Life Sciences, Bar-Ilan University, Ramat-Gan, Israel.

Corresponding author:

*Stefano Goffredo (s.goffredo@unibo.it)

Table

Supplementary Table S1. Number of analyzed polyps (n) in the four Sites during the gonadal development and fertilization periods. For each Site, the reproductive state (Female, Male and Sexually inactive) and embryogenetic females of the analysed polyps is reported.

Gonadal development period

Site	pH_{TS}	n	Female	Male	Sexually inactive	Embryogenetic
1	8.07	18	7	7	4	0
2	7.87	12	5	6	1	0
3	7.74	12	7	4	1	0
4	7.40	19	4	7	8	0
Total		61	23	24	14	0

Fertilization period

Site	pH_{TS}	n	Female	Male	Sexually inactive	Embryogenetic
1	8.07	17	5	3	9	3
2	7.87	18	4	5	9	2
3	7.74	15	2	10	3	1
4	7.40	14	4	6	3	2
Total		64	15	24	24	8

Figure

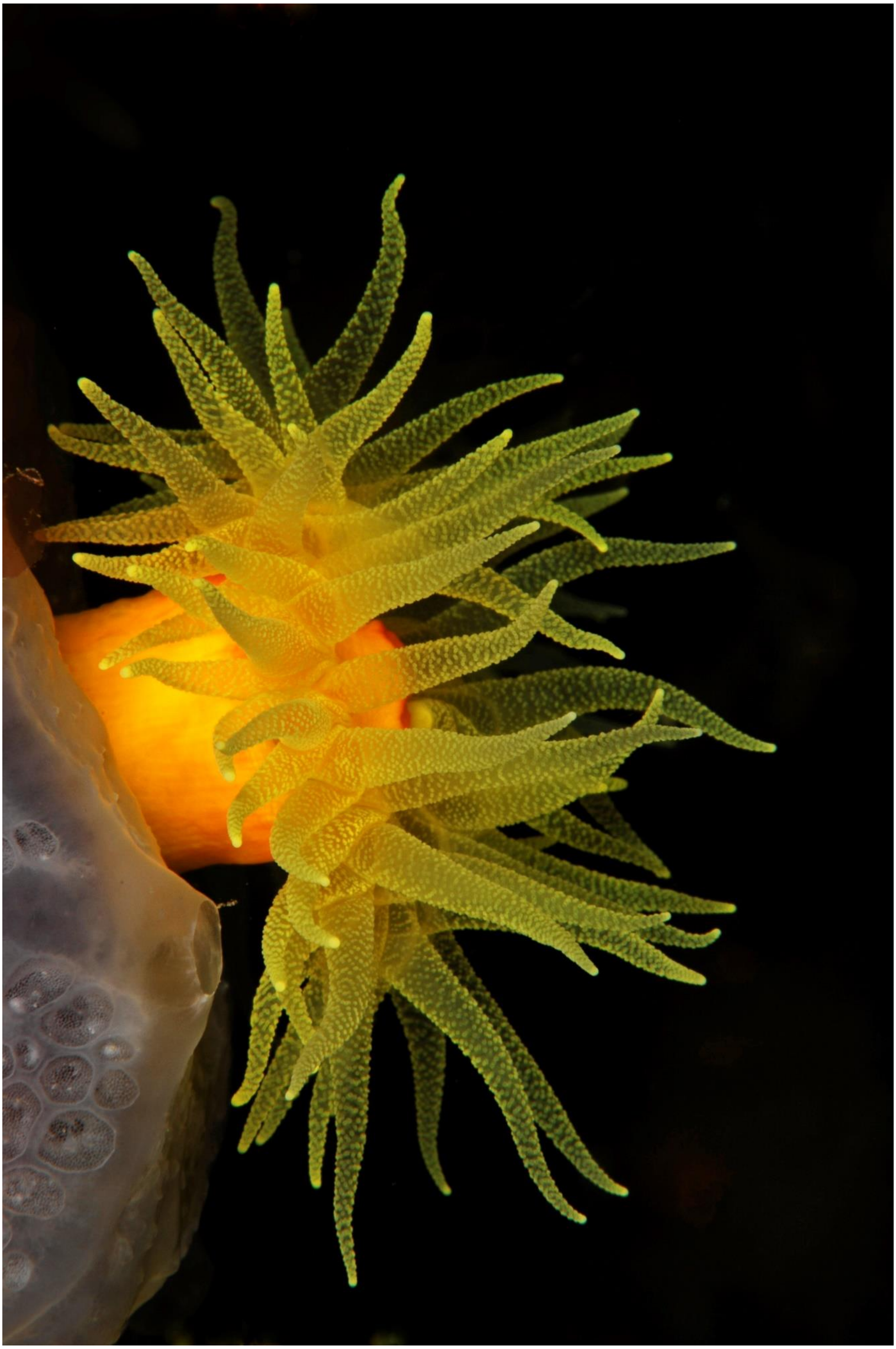

Supplementary Figure S1. Living specimen of *Leptopsammia pruvoti* in Pietra Nave. Photo by courtesy of Francesco Sesso.
